# The Hospital. Nursing Section

**Published:** 1905-03-25

**Authors:** 


### The Hospital.

### Nursing Section.

Contributions for this Section of "The Hospital" should be addressed to the Editor, "The Hospital," Nursing Section, 28 & 29 Southampton Street, Strand, London, W.C.

No. 965.—Vol. XXXVII. *SATURDAY, MARCH* 25, 1905.

### Notes on News from the Nursing World.

### SCARCITY OF NURSES IN MANCHURIA.

It will be recollected that when the war in the Far East broke out we made inquiries at the Russian Embassy here as to whether outside help was likely to be required by the Russian Army in the care of the sick and wounded. The reply was that no information on the subject had been received from St. Petersburg, but that there was reason to believe that there would be no difficulty in obtaining an adequate supply of Russian nurses. This, however, was at the beginning of the campaign; but now, according to accounts from Harbin, there are only 60 doctors and 140 nurses to look after 50,000 wounded and 12,000 sick soldiers located in that town. Many of the unfortunate creatures are still lying in the cars and trucks in which they were brought into Harbin. In these circumstances it is not surprising that the deaths during last week were upwards of 5,000. If there had been a sufficient number of doctors and nurses there is no doubt that this number would have been materially reduced.

### GENERAL TRAINING AND MIDWIFERY.

In the interview which our Commissioner had the other day with the matron of the City of London Lying-in Hospital, she expressed the opinion that all girls who propose to be midwives should previously take general hospital training. Miss Fox states that in her present position she has found her own general training to be of the utmost value, and it is obvious that if all midwives were also fully-trained hospital nurses the elevation of the entire class would follow as a matter of course. In time to come this ideal may be realised, and even in the case of hospital-trained nurses who have no idea of practising as midwives, it may be found worth while to qualify for the work.

### QUEEN'S NURSES IN ELEMENTARY SCHOOLS.

A meeting of the Council of Queen Victoria Jubilee Institute for Nurses was held last week. The reappointment of the Council for the ensuing three years by the Queen having been reported, Mr. Harold Boulton was re-elected honorary treasurer, and Mr. W. G. Rathbone honorary secretary. The various committees for the year were then appointed, after which a discussion took place as to the advisability of school nursing being undertaken by Queen's nurses in the elementary schools. It was ultimately agreed to make a representation to the Board of Education pointing out the advantages to the children and to the community of the work being carried out by Queen's and other fully-trained nurses. We trust that the Board of Education will recognise the force of the argument in favour of this course. School nursing is, in fact, far too important to be entrusted to persons who have not been adequately trained.

### SCHOOL NURSES IN NEW YORK.

How valuable the work of nurses in the schools of New York is, may be gathered from the record of last year. The total number of children under supervision in the schools was 318,688, and of these 41,619 were treated individually. The staff of nurses numbered 35, but since the opening of the present year it has been increased to 42. A new departure has been made with the view of ensuring that the eyes of the children shall be cared for more effectively. The medical inspectors make tests for refraction, and where the sight is in any way impaired, a card is sent to the parents informing them that the vision of the child is defective and recommending that it should be sent to an oculist or to one of the eye hospitals. In addition to this the nurse visits the home and explains the necessity for immediate treatment. A large percentage of children have been found who needed glasses, and the parents readily avail themselves of the advantages offered.

### THEORETICAL TRAINING IN HOUSEHOLD MATTERS.

The subject of preparatory training for nurses is engaging a good deal of attention in Melbourne. Miss E Glover, who notes that the American idea of preparatory training includes anatomy, physiology, cookery, household economics, English language, and vocal expression, asks how all this can be done in six months, and what good so much theoretical teaching can possibly be in household matters. Her view is that an ounce of practice is worth a pound of precept in cookery and household economics. She goes on to contend that domestic work is the foundation upon which nurses must build if they mean the nursing edifice to stand against the storm of criticism just now waged against it, and she insists that the girl who proposes to become a nurse should first of all be a proficient in household matters. Miss Glover acknowledges the great educational advantages enjoyed by nurses of the present day, but she thinks that there is another side to the picture. "We get a nurse who can expound at any length on the chemistry of food, but who cannot cook a dinner for her patient; we get a lady who can eloquently point out the evils of a dirty room, but who would be worn out if she had to put that knowledge to a practical use by cleaning it." All this may often be true in respect to nurses here as well as nurses at the Antipodes; and we agree with Miss Glover as to the value of preparatory training. But in six months an ordinarily intelligent girl should be able to learn a good deal about cookery and household matters, even if she did not acquire proficiency in the other subjects.

### NURSES AND "BEER MONEY."

The only satisfactory way of dealing with the question of beer money, which was discussed at the meeting of the Metropolitan Asylums Board on Saturday, is to abolish it. The Board have decided to make the matter the subject of special inquiry, but so far as nurses are concerned, at any rate, there is nothing to be said in favour of the maintenance of a custom which at some archaic period may have been defensible, but is now both incongruous and unpopular. If we are not quite prepared to endorse the assertion of a member of the Asylums Board that it is "disgraceful" that respectable girls, many of them ladies, acting as nurses in the hospital, should be offered beer money, we do not believe that any evidence to justify the retention of the practice can be forthcoming at the inquiry. The sum allowed to nurses in lieu of ale, which they do not wish to drink, might be added to their salaries.

### BRIGHTON GUARDIANS AND QUEEN'S NURSES.

At the last meeting of the Brighton Guardians an application from the Brighton branch of the Queen Victoria Jubilee Institute for an increase in the annual contribution of the guardians was discussed. The present amount is £25, and it was pointed out, in support of the application, that Cardiff Guardians contribute £63, Blackburn £100, Bolton £105, and Gloucester £150 to a similar object. It was also mentioned that in 1904 a total of 41 out-door Poor-law patients were attended by the Queen's Nurses in Brighton, the number of visits paid being 1,168. A motion to increase the contribution from £25 to £40 met, however, with a great deal of opposition, and on a division it was defeated. We think that the Brighton Guardians came to a mistaken conclusion. Brighton, as one of them urged, is wealthy, but that is hardly the question. The question is whether, or to what extent, the work of the Queen's nurses tends to keep down the rates. It would probably be found that a contribution of £40 would not mean any addition to the expenditure of the guardians.

### THE PERIL OF LARGE DONATIONS.

At the annual meeting of the Taunton District Nursing Association, the gratifying announcement was made that the receipts had more than covered the expenditure. This means that the Association is out of debt. It was also, however, intimated that the amount received from annual subscribers was supplemented by a couple of handsome special donations. These, of course, were very acceptable, but the chairman rightly reminded the members that when the financial position of an organisation is improved by exceptional causes, there is a danger lest the public should imagine that it can dispense with much of the external assistance it has been accustomed to receive. As far as possible, donations should be appropriated to the purpose of building up a reserve fund.

### CHANGES IN THE MILITARY NURSING SERVICE.

We are officially informed that Miss M. J. Hepple and Miss M. B. Williams have been appointed staff nurses in Queen Alexandra's Imperial Military Nursing Service; also that Staff Nurse E. M. Lang has been posted to the Station Hospital, Lincoln, for temporary duty; that Sister C. G. Stronach and Staff Nurses E. Barber, W. M. Jay, M. MacGregor, and B. F. Perkins have been warned to prepare for service abroad; that Sisters D. V. Briscoe and D. I. Rickards have resigned their appointments on account of their marriage; that Sister A. Guthrie has been transferred to South Africa from the Guards' Hospital, London, Sister W. Potter, to the Royal Infirmary, Dublin, from the Royal Herbert Hospital, Woolwich, Sister M. L. Potter, to the Royal Herbert Hospital, Woolwich, from the Royal Infirmary, Dublin, in exchange with Miss W. Potter, Sister C. K. E. Steel, to South Africa, from the Guards' Hospital, London, and Sister B. S. Vaughan, to South Africa, from the Station Hospital, Chatham.

### MISS WEDGWOOD.

At the annual Court of Governors of the Royal Free Hospital last week the Princess Christian said that she wished, as President, to add her testimony to the sorrow and grief which they all felt at the loss of Miss Wedgwood. The Princess added, "She is a very old friend of mine, and I am sure that we shall miss her services very much." The Governors have sent a silver salver and an address to Miss Wedgwood.

### PENSION FUND MEETINGS IN SCOTLAND.

The Chairman of the House Committee of the Victoria Infirmary, Glasgow, has kindly given permission for an address to be delivered at that institution on Wednesday the 29th instant, at 4 P.M., on the subject of the Royal National Pension Fund for Nurses, by the secretary, Mr. Louis H. M. Dick. The address will be of an informal character, and, it is hoped, will be followed by discussion. The managers of the Royal Infirmary, Edinburgh, have also been good enough to allow a similar meeting on Thursday, 30th inst., at 5 P.M. With a view to making these gatherings representative of Scottish nursing, matrons, superintendents, and nurses of other institutions have been invited to attend.

### NURSING ON A FLOATING HOSPITAL.

We publish this week the second and concluding portion of an article by Miss Wilber, superintendent of nurses at the Boston Floating Hospital. The unique character of the hospital, which, with characteristic enterprise, the Americans started ten years ago, in order to afford infants suffering from gastro-intestinal and other non-contagious diseases, the best of modern medical and nursing care, was fully described in the first instalment. To-day Miss Wilber describes the work of the nurses, and refers to the post-graduate course for those desiring to study the care and diseases of infants. None but nurses who have received two years' general training can be admitted to the course, which is followed by an examination. The details of last year's examination are given by Miss Wilber, who also enumerates the special rules which are impressed upon every nurse who enters. They are of a very stringent character, and their strict enforcement is clearly essential in order to maintain the usefulness of the Floating Hospital.

### A RESIGNATION AT LEAVESDEN.

At a meeting of the Metropolitan Asylums Board on Saturday last, the Finance Committee reported that, owing to failing health, Miss Cottrell had resigned her appointment as assistant matron of Leavesden Asylum, and claimed her superannuation allowance. The committee stated that Miss Cottrell had held her present appointment continuously for the past 26 years, and they had considered a proposal from the Asylums Committee that four years should be added to her actual period of service, for the purpose of computing the amount of her superannuation allowance. The committee concurred in in the proposal, and the recommendation to that effect was agreed to.

### THE MIDWIVES ACT IN DEVONSHIRE.

The Devonshire County Council have delegated the administration of the Midwives Act to such District Councils as will accept the delegation of duties, and where they have declined they have placed the work of notification and supervision under the Medical Officers of Health. Despite extensive advertising very few midwives have registered in Devonshire, and many County Councillors being of opinion that the registration fee is too high, the County Council have decided to join the East Suffolk County Council in representing that view to the Central Midwives Board.

### BEQUESTS TO NURSES.

The will of Mr. James Douglass, of Down Street, Piccadilly, which was proved the other day, is notable for the fact that he bequeathed all his furniture and a sum of £500 to the nurse who attended him in his last illness. The bequest, it is stated in the will, was made in grateful recognition of the unvarying care shown by the nurse to her patient. Mr. Douglass also left £250 to a nurse who attended him during an illness in 1900. As his estate was valued at the gross amount of £23,000, it cannot be said that the nurses benefit unduly at the expense of relatives.

### SUICIDE OF TWO GERMAN NURSES.

On Friday evening, two young women attired as hospital nurses entered the Kurfürsten Hotel, Berlin, and asked for a room. They arrived in a carriage, and, though they had no luggage, a room was assigned to them out of respect for their uniform. As they did not make their appearance on Saturday morning, the hotel authorities became anxious and sent for the police, by whom the door of their room was opened. It was then found that both the girls had committed suicide by taking morphine, life in each case being quite extinct. A letter, which had been placed by the bedside, stated that they were two Sisters at the Municipal Hospital, Charlottenburg, but no reason appears to have been given for the terrible act.

### MORE NEW SUBSCRIBERS AT STOCKTON.

The report of the Stockton and Thornaby District Nursing Association, which was adopted at the annual meeting, brought out the interesting fact that as the result of the issue of a special circular letter last year appealing for additional help, 292 new subscribers were secured. This is a substantial result, and should encourage the members of the Council to make a further effort in the same direction during the present year. For, as the expenditure of the Association in 1904 exceeded the income by nearly £100, and the balance at the bank has been consequently reduced almost to vanishing point, it is essential that the income should be considerably augmented. The nurses last year paid nearly 1,500 more visits than in 1903, thanks to an increase in the staff, which accounts for the higher expenditure; but it would, of course, be impossible for the Association to continue indefinitely to employ more nurses than the financial support justifies.

### YORK HOME FOR TRAINED NURSES.

The report presented at the annual meeting of the subscribers to the York Home for Trained Nurses, shows an increase of 79 cases, or 466 in all. Attention is called to the fact that the entire work of the home has been carried on by 56 nurses, and that to meet exigencies there are 19 probationers undergoing training at various hospitals. Fifteen of these are being trained for three years. It appears that the York nurses are in general request. During 1904 they were engaged for private cases in the counties of Berkshire, Cambridge, Durham, Gloucester, Huntingdon, Lincoln, Northumberland, and Sussex, also in Wales and Scotland, as well as in the city and county of York. As to institution work, they were engaged in three Poor-law infirmaries, in five cottage hospitals, and in eight isolation hospitals; while district work was undertaken in the country districts of Wetherby, Boroughbridge, Burton Spa, and Driffield. With regard to the district nurses no fewer than 507 among the sick poor in York were attended by them, and on this ground the home claims the support of the charitable. We observe with pleasure that during the entire year the sister superior did not receive a single complaint from patients or their friends or medical attendants as to the manner in which the nurses perform their duties.

### SHORT ITEMS.

The Nurses' Registration Bill, which was the third order of the day on Friday in the business of the House of Commons, was not reached.—The sister in charge at the Drill Hall, Lincoln, informs us that Messrs. Edward Cook and Co., Limited, have sent a large quantity of their excellent soaps as a present for the use of patients suffering from typhoid fever, and that she is most grateful for their kindness.—The death is announced in Scotland of Miss Mary J. Robson, formerly matron of the Hospital for Skin Diseases in Fitzroy Square, London.—The many friends of Mrs. MacIntyre, matron of Sir Julian Goldsmid's Home of Rest for Nurses at Brighton, will be interested to hear that a testimonial fuud has been started in order to present her, on her resignation, with a token of the appreciation in which she is held. Subscriptions should be sent to Miss Lilian Brooke-Hunt, Albert Gate Court, 124 Knightsbridge, S.W.

### The Nursing Outlook.

"From magnanimity, all fear above;
From nobler recompense, above applause,
Which owes to man's short outlook all its charm."

### PRACTICAL NURSING DIFFICULTIES.

The position of nursing affairs in Great Britain to-day is by no means clear to the majority of people. This is natural, for the public assume that the great nurse-training schools should know their business, and that they ought to voice nursing opinion and to make it their first aim to protect the public interests as well as those of the nurses and themselves. It is however beginning to dawn on the public that for some reason which to them is unexplained the nurse-training schools have been passive resisters to every proposal which aims at co-operation and a practical solution of the many difficulties which have arisen in the field of nursing. This state of affairs is no doubt due to the anomalous position which is taken up by the nurse-training schools. They are vigorously opposing the bills before Parliament for the State registration of nurses, which in fact do not recognise the nurse-training schools or provide that they shall be represented. This opposition is natural in the circumstances, but the same cannot be said of the recent decision of the majority of the representatives of the schools to petition the Board of Trade not to grant a license to the Incorporated Society for Promoting the Higher Education of Nurses. It is regrettable that the training schools have invariably presented a *non possumus* attitude to every proposal which aims at a solution of the present difficulties, whilst they steadily refrain from co-operating together and suggesting a remedy of their own to the existing evils. Who can wonder if the nurse-training schools should soon cease to carry weight in nursing affairs in Great Britain, as their policy is compelling the public to regard them as private adventurers, each considering their own interests as paramount?

Again, it may be interesting to show that in the United States great difficulties have arisen in regard to nursing matters which still await solution. It appears that the States in which there have been legislative enactments for the registration of trained nurses are New York, New Jersey, Virginia, North Carolina, and Maryland. It is too soon probably to give an authoritative opinion as to the effect of these Acts upon American nursing, but so far they appear to have effected little. None of these Acts prevent the continuance of the work of nursing by persons who do not assume to be "registered nurses." In Massachusetts a great battle is at present being fought which well illustrates the difficulties presented by the problem in certain States. A Bill for the registration of nurses has been before the Massachusetts Legislature on petition of the State Nurses' Association, and a great assemblage of nurses, with some physicians and hospital trustees appeared before the judiciary committee, when considerable opposition was developed on the part of the small and special hospitals, especially for the insane. The Act as originally drawn practically excluded the latter, but the promoters brought up a series of amendments which met many of the objections, and by liberal concessions satisfied the majority of their opponents. In the Massachusetts Bill the promoters insisted upon the qualification of not less than two years in a hospital. This provision excludes the Waltham plan, and many small hospitals with successful schools, on the reasonable ground that the latter system assumes to train nurses in the homes of patients and in district nursing in such a way that the pupil nurses may not get two full years of service in a hospital. The promoters of the Bill insist that the Waltham plan must come up to the requirement of two years' actual hospital work, and on this point we entirely agree with the promoters.

Another point of objection which has much force in it has been raised to the proposal that the Registration Commission under the Bill shall be wholly composed of graduate nurses, which would exclude entirely members of the medical profession who are teachers and trainers of nurses, as well as the trustees of hospitals who provide the funds for such training. Hospital and medical opinion favour the inclusion of representatives of the latter upon the Commission, and we incline to think that the promoters will be wise to yield such representation by admitting two medical men on the Commission of five, who will have control of State registration in Massachusetts. Such a decision would not only be just to the training schools, but it must increase the authority and influence of the Commission, which would thus secure certain advantages politically that might prove useful in the conditions which are likely to arise in regard to future legislation. We understand that a final decision is not likely to be reached during the present session of the State legislature, but there is every reason to hope that the present discussion in Massachusetts will result in a better and wiser solution of the difficulties presented than has yet been attained anywhere in the United States.

In any case, the fuller the knowledge and the more mature the judgment of those who bring their minds to bear upon nursing matters in Great Britain to-day, the more certain is it, that they will be forced to the conclusion, that the present attitude of the nurse-training schools cannot be maintained, and that united action on their part and a definite lead is urgently called for. Whether that lead should come through the Central Hospital Council, or by conference with the promoters of the Incorporated Society for the Higher Education of Nurses, or in some other way, is open to debate at the moment. The means whereby united action can be secured are relatively unimportant, provided the definite and continuous co-operation of all the higher authorities charged with the duty of training nurses in Great Britain to-day can be secured. The attainment of this object should unite all who are genuinely interested in the welfare and progress of nursing matters in this country at the present time.

### Lectures upon the Nursing of Infectious Diseases.

By F. J. Woollacott, M.A., M.D., B.S.Oxon, D.P.H., Senior Assistant Medical Officer, Park Hospital, Metropolitan Asylums Board, Hither Green.

### LECTURE XVII.—ENTERIC FEVER.

Enteric or typhoid fever is an acute infectious disease, usually of considerable duration and attended by grave symptoms. Like many others of the infectious diseases it is most prevalent during the autumn and early winter. From time to time it breaks out in epidemic form, but in consequence of the improvements in sanitation, such epidemics are rarer and less severe than formerly. Whenever a large number of people are crowded together and obliged to live under unhealthy conditions, it is almost certain, sooner or later, to show itself. This is particularly the case with armies in time of war, and the ravages of enteric fever among our own troops during the recent South African War will be fresh in the memory of all. Until a few years ago its cause was not clearly understood, although it was realised to be in some way or other connected with filth and overcrowding, but we now know that it is due to certain microbes known as typhoid bacilli, which, under the microscope, may be seen moving about like a number of very minute worms. The bacilli escape from the body of the patient in the discharges, chiefly the stools and the urine, and, if these are properly treated and disinfected, the spread of infection can be prevented. Consequently it is rare for enteric fever to spread directly from person to person if suitable precautions are observed.

The usual path by which infection is conveyed is water, to which the germs have by some means or other obtained access. This is commonly due to contamination with sewage, and if such water is drunk or used in preparing food, *e.g.* in washing vegetables, or if oysters and other shell-fish or watercress taken from polluted waters are eaten, the disease is likely to result. Milk, too, has been responsible for many outbreaks, usually because infected water has been used to wash out the milk cans. It should be remembered that any possible danger from either milk or water may be removed by boiling, and, if they are not quite above suspicion, this should always be done, for the germs are readily killed by exposure to a high temperature.

The typhoid bacilli and the poisons produced by them may cause more or less mischief in every part of the body, but the most characteristic and important changes are found in the abdomen. The lower part of the small intestine contains certain glands, collected into groups known as Peyer's patches. Each patch is situated in the mucous membrane, just beneath the surface, and is from one to three inches long, and about half as wide. In the large intestine are similar but smaller collections of glands. It is on these Peyer's patches, and to a less extent the glands of the large intestine, that the chief stress of the disease falls. Certain of them, sometimes a few only, sometimes many, become inflamed and swollen, the mucous membrane covering them gives way, and after a time, usually from two to three weeks, they lose their vitality and come away as sloughs, which may be found in the stools if careful search is made. Well marked and often extensive ulceration is left, which, in favourable cases, gradually heals as the disease subsides. It is important to keep these intestinal changes constantly in mind, for they will be found to explain many of the symptoms and complications of enteric fever, and also furnish a guide to the appropriate treatment. The inflammation of Peyer's patches is accompanied by swelling of the lymphatic glands in the abdomen and enlargement of the spleen.

Enteric fever shows a special tendency to attack older children and young adults, and, though no age is exempt, the majority of patients are between the ages of ten and thirty. In young children the attack is usually comparatively mild.

The incubation period varies considerably, from one to three weeks, a fortnight being perhaps the most common time. The onset of the disease is nearly always insidious, with vague and indefinite symptoms. The patient feels out of sorts, loses his appetite and is easily tired. He grows steadily weaker and in a few days has to take to his bed. There is usually more or less headache, with pains in the limbs and sometimes bleeding from the nose. In other cases the onset is more acute and marked by vomiting, abdominal pain, diarrhœa, or perhaps shivering attacks; or the true nature of the disease may be obscured by the early appearance of some complication such as pneumonia. At times the patient is able to struggle on, doing his ordinary work, till the disease is well advanced, and may not seek medical advice until thoroughly prostrated or until some complication has shown itself.

The further course of the illness may vary considerably both in severity and in the nature of the symptoms. The following is a brief account of a typical case of average severity and free from complications. During the first few days the temperature rises steadily, with partial morning remissions, until it may reach 104° F., or even more. The patient sleeps badly, suffers considerably from headache, and may be delirious. The tongue is furred, the appetite lost, and there may be diarrhœa and some abdominal pain and tenderness. As a rule there is more or less cough. At the end of a week the characteristic rash begins to appear. It is usually very scanty, and consists of small pink or red spots, slightly raised, and just perceptible to the touch. They come out in crops, each lasting for a few days, and then gradually fading. They are chiefly found on the abdomen and back, but occasionally on the rest of the trunk, and even the limbs. Frequently their number is very small, only two or three being present at any one time, and, as they are very inconspicuous, they may easily escape notice unless carefully searched for. During the next week or ten days the fever is at its height. The temperature remains high, with only slight morning remissions, and the patient becomes more and more prostrate. He is often dull and apathetic, and usually somewhat deaf. Delirium is common. The tongue and lips are dry, and tend to become brown and cracked. In the absence of proper nursing, dried mucus and particles of food collect round the gums and teeth, and form brownish masses known as "sordes." The abdomen is distended, and there is frequently diarrhœa, the stools being semi-fluid and pale yellow, resembling pea-soup in colour and appearance. The pulse is weak and rapid, and the patient's whole condition is one of extreme feebleness. At the end of this stage, in favourable cases, signs of improvement appear. The temperature begins to fluctuate, showing more and more marked remissions each morning. The evening temperature, too, gradually falls, and in the course of a week or ten days becomes normal. At the same time the patient's general condition gradually improves. His mind grows clear, and interest in his surroundings returns; the tongue and mouth become clean and moist, and the appetite returns; the diarrhœa ceases, and the stools resume their normal appearance. The convalescent stage is usually somewhat prolonged and tedious, for the patient has been so weakened by the disease that it is a long time before he regains his usual strength.

Such is the course of a typical, uncomplicated attack of enteric fever. Frequently, however, especially in children the symptoms are milder and of shorter duration. In such cases the temperature is rarely very high, and begins to drop at the end of the second week, or even earlier. The diarrhœa is not excessive, and may be altogether absent, and the patient, though weak, is not seriously prostrated. As a rule he soon regains his strength, and convalescence is rapidly established. It is important to bear in mind that these comparatively mild cases should be treated with the same care and watchfulness as are the more severe ones, for there is always the possibility that complications of the most dangerous character may arise.

On the other hand, an attack of enteric fever may assume much more severe characters. The symptoms already described become aggravated, and others make their appearance. The temperature keeps high, and the patient very rapidly loses strength, and is soon entirely prostrated. Owing to the intensity of the fever he quickly becomes emaciated and very tremulous. There is constant muttering delirium, and the diarrhœa may be profuse and exhausting. In the worst cases he may drift into a state of semi-consciousness. He then lies helplessly in bed, quiet, except for restless movements of the hands, and quite oblivious to what is happening around him. The urine and fæces are often passed involuntarily. This condition is known as the "typhoid state," for it is more common in typhoid fever than in any other disease. There is always the gravest danger, for at any moment complications may appear, or death may result from exhaustion and weakness of the heart. As might be expected, in these severe cases the process of recovery is always very slow. One of the first signs of improvement is some remission in the morning temperature, followed by a gradual diminution in the severity of the symptoms. Some weeks usually elapse before the patient is able to leave his bed, and convalescence is often hindered by more or less serious complications.

Unlike most other of the infectious diseases relapses are comparatively common in enteric fever. As a rule a relapse begins within a few days after the temperature has fallen to normal. The course and symptoms are precisely the same as those of the primary attack, but, on the whole, are of a milder character and shorter duration. Recovery follows in the great majority of cases, but death may result, usually in consequence of some complication. Occasionally there may be two, or even three, relapses. During a relapse fresh ulcers form in the intestine, some of the Peyer's patches becoming inflamed, and sloughing in the manner previously described.

### The Boston Floating Hospital.

By L. A. Wilber, Superintendent of Nurses.

(*Concluded from page* 324.)

The nursing on the Boston Floating Hospital has always been of increasing interest. During the first few years when only day patients were treated the nursing was done largely by volunteers, graduates from the large hospitals, who did not happen to be busy. As soon as trips were made every day volunteer service was not sufficient, and when we began to care for patients at night as well as in the daytime, an adequate nursing force was a necessity.

### Summer School for Nurses.

With the growing love and appreciation of out-door life have come out-door hospitals of various kinds—tent service connected with large hospitals and floating hospitals. Also in connection with out-door life has come the summer school, where, for a few weeks, those too busy during the rest of the year can perfect themselves along special lines. On the Boston Floating Hospital there is the summer hospital and the summer school in combination. Here, for ten weeks, there is established a post-graduate course for nurses desiring to study the care and diseases of infants. Theoretical study and practical work are combined as in most schools for nurses. The work is hard and it is no vacation, but the experience gained is of much value. The requirements for admission to this course are that the nurses must have received a two years' training, and must be able to furnish satisfactory references. The theoretical training is given by physicians, specialists in children's diseases, in the form of lectures, notes of which are handed in for correction. After all lectures have been given comes a series of quizzes on subject matter of lectures, conducted by the superintendent of nurses, or one of her assistants; these are for the purpose of review before the examinations.

### Hours of Duty in the Wards.

The wards are in charge of head nurses who have taken the course during previous seasons and under their guidance the practical work is done. This includes care of some of the most severely ill babies from the city or suburbs, giving of baths of various kinds, hot, warm, cold, salt water, oil, sponge, fan, hot or cold pack, etc., the use of various foods and different methods of feeding—bottle, spoon, dropper, Breck feeder, œsophageal tube, or nasal tube. Special treatment charts are kept for each case, noting food and medicines given and effects noticed, special note being made of character of dejecta to enable physician to determine where stress of disease falls in the gastro-intestinal diseases, which comprise a large percentage of the cases. Mechanical methods of treatment, such as washing of stomach, irrigation of colon, treatment of emergencies which often arise, observation of pulse and temperature, with feedings every two hours, all combine to make up a very busy day, which is also a long day, as in most hospitals, the nurse going on duty at 7 A.M. and remaining until 8 P.M. The nurses have one afternoon from 4 P.M. weekly, and one hour daily off duty, this latter being spent on the upper deck aft in a portion curtained off for their use and fitted up with reclining chairs. This place is also utilised by the night nurses after dinner, before going on duty for the night, in which they can read, write or rest, as they please, and at the same time enjoy the ever-changing scenes of the harbour.

### Special Rules of the Hospital.

Certain peculiar rules of the hospital are impressed on the nurse. Recognising the infectious nature of the cases and the added danger of infection because of the large number of patients and the small amount of room, as is necessarily the case on a boat, it has been found necessary to observe, special precautions, such as careful scrubbing of hands and forearms in antiseptic solution after any possible contamination, and always before feeding the babies, and before the nurse goes to her own meals. The wearing of elbow-sleeves for both doctors and nurses is the rule, and an added protection is the wearing of an outer or surgical apron, which is removed when not actively on duty in the wards. Flies are carefully excluded from the wards. An important rule for the protection of the babies is the care of the nipples used on the feeding-bottles; these, besides being thoroughly washed, are boiled for a few minutes before each feeding;

### THE BOSTON FLOATING HOSPITAL—*Continued.*

then only the nurse on duty in the food department, with specially scrubbed hands, is allowed to touch them. If when given to the baby the nipple needs readjusting, the bottle is taken back to the food department to have it done there.

### The Milk Used.

The necessity of a pure, fresh milk for the babies is emphasised, the milk used being from the Walker-Gordon farm, a firm well known for the extra precautions in the way of cleanliness which they take to prevent contamination of milk by bacteria. The milk is delivered on the boat about four hours after milking, in quart glass jars packed in ice. This milk is so free from bacteria, and so pure and so fresh, that it is not considered necessary to pasteurise it. Each nurse receives instructions in preparing modified milk, and demonstrations are given in the food department regarding care of milk, temperature at which it should be kept to prevent growth of bacteria, and the necessity of sterilisation of all utensils used in making the modifications in order to prevent contamination.

Occasionally the nurses have opportunity to observe the diseased tissues of fatal cases, and thus they learn why their untiring efforts have not been successful. Those nurses whose work is satisfactory, and who attain 75 per cent. in examinations, are given the diploma of the school.

The following were the subjects of the lectures last year:—Peculiarities in Anatomy and Physiology of Children; Care of Premature and Sickly Infants; Management of Digestive Disorders and Infants' Feeding; What to Observe in Children; Diseases of the Gastro-enteric Tract; Congenital Defects and Deformities; and Congenital Syphilis.

### Nurses' Examination.

The members of the Post-Graduate School were examined in the following subjects:—

1. What points should be noticed about the intestinal discharges of infants?

2. (*a*) Describe in detail the preparation of a day's food for a well child to have six feedings of 8 oz. each, made up in the proportion of about 8 parts of top milk, 10 parts of boiled water, 3 tablespoonfuls of milk-sugar, and 10 per cent. of lime-water. (*b*) State supplies necessary, how they are to be made ready, and how kept, including apparatus.

3. Compare the relative development of the head, thorax, and abdomen in the child at birth and at the age of five years.

4. Give some account of three affections liable to appear in the new-born child.

5. Describe the dejections in fermental diarrhœa and in ileo-colitis.

6. (*a*) What substitutes for milk are used in a case of ileo-colitis? (*b*) Give full details for preparing one of them.

7. Give the treatment of umbilical hernia.

8. What would you do if you were placed entirely on your own resources in regard to the child in an impending premature delivery?

9. What are the characteristics of the pulse and respiration in childhood?

10. Tell what you know about the excretion of urine in the child.

### The Pupils at the City of London Lying-in Hospital.

INTERVIEW WITH THE MATRON. BY OUR COMMISSIONER.

The fact that the Midwives Act comes fully into operation on the first of April invests institutions at which midwives are trained with special importance at the present moment. The City of London Lying-in Hospital is, of course, a school for midwives as well as for monthly nurses, and naturally Miss A. M. Fox, the matron, takes a keen interest in the working of the Act.

"It has not, however," she said on the occasion of my visit to the institution in City Road, "so far affected us, except that the pupils are required under its provisions, to deliver 20 cases under the supervision of a doctor or qualified midwife, instead of 5 as required by the London Obstetrical Society. They must also see cases right through. We were recognised immediately as a school by the Central Midwives Board, and we have taken care since the Act was passed to fulfil its conditions to the letter."

"Has it tended to either increase or diminish the number of your pupils?"

"Perhaps the best answer to that question is that we could have twice as many pupils as we are training now if we had accommodation for them and a sufficient number of cases."

### Working under Drawbacks.

"Will there be more space when the new hospital is built?"

"There will be 52 beds instead of 42. But in the existing state of affairs we work under very considerable drawbacks. In addition to the inconvenience of the temporary hospital being on one side of the road and the nurses' quarters on the other, I suppose that the cost of carrying on the work thus is quite £1,000 a year more than when we were in the old building. It is now 18 months since the warehouse in Old Street was adapted for a hospital, and I anticipate that it will be a year and a-half more before the permanent one is finished."

"You have a fine site at the corner of the four roads?"

"Yes; but the site would not have been cleared if the committee had not found that the construction of the tube railway had so seriously damaged the foundation and structure that the original building could not in any circumstances be safe. Accordingly, instead of altering and improving it, they decided to do away with it. A new lying-in hospital has not been built for about a century. Ours will be on the most modern lines, and, we hope, the best of its kind. There has been considerable delay in commencing the new building, in consequence of the tenders far exceeding the amount estimated and therefore having to be revised. Meanwhile, the work, in spite of all the disadvantages, has been going on just the same, with as many cases and as many pupils."

### The Conditions of Admission.

"That is a matter for congratulation, and it must also be some consolation to you that you are, at any rate, provided with excellent quarters for the pupils."

"We are certainly well off in that respect, though we shall be very glad when the pupils are close to the wards. Each pupil has a bedroom to herself, and, as you have seen, there are spacious sitting and dining-rooms."

"At what age do you admit the pupils?"

"From 21 to 45. I said just now that we should need more cases as well as space in order to take more pupils. Our average number is 30, of whom 18 are monthly nurses and 12 midwives, and unless the cases increased we could not exceed it."

"What are the periods of training and the fees?"

"The course of training for monthly nurses is two months, and the fees are 12 guineas; for midwives the course is four months, and the fees are 28 guineas. Since the Midwives Act passed, we have taken some pupils for 22 guineas and given them two months' training in monthly nursing and one in midwifery, but this does not entitle them to register as midwives. Very few of the pupils who train here want to practise as midwives, because they know that they can make more money as monthly nurses."

THE EARNINGS OF MONTHLY NURSES.

"Notwithstanding the fact that they study for midwifery?"

"Yes. As a matter of fact, monthly nurses earn more money than either midwives or the majority of nurses who have been trained at general hospitals. Many of our former pupils think that they are badly done by if they are only paid two guineas a week. Yet it is a mystery to me where they all get work."

"I suppose they come to this hospital from various parts of the country?"

"From various parts of the world. There are two here just now from Australia. We have had numerous pupils from South Africa, several from India, Egypt, and America."

"But surely there are plenty of good training schools in America."

"There are supposed to be plenty. But the pupils who come here from the States, and from the Antipodes, say that English certificated nurses can always get work."

"How many pupils of both classes did you have last year?"

"A hundred and seventy."

DAY AND NIGHT DUTY.

"The hours on and off duty, I conclude, differ from those of a general hospital?"

"Very considerably. For example, the pupils do not ordinarily begin work until 8.30 A.M. Then they have no set time for off duty. They can always go out when they are not wanted. But they do not wish to go out much because the time of training is so short. Moreover, if they are out they may lose something that is interesting. They like seeing all the deliveries because it is a great help to them afterwards."

"Is there much night duty?"

"There are always four on night duty, and each pupil usually has a fortnight of night work. They learn a great deal while they are on night duty."

OUTSIDE WORK.

"The training, I suppose, is partially in the wards and partially outside?"

"Two months in the wards and two months outside. There are four wards in the temporary building, and unless pressed for room we only put eight patients in each. But here are figures which will give you an idea of the proportion of cases. Since the hospital was established in 1750, over 64,000 women have been delivered here. And since the out-patient department was instituted in 1872, no fewer than 45,000 have been delivered in their own homes."

"The area of work must be very large?"

"We have eight outside registered midwives, and their centres are Islington, Clerkenwell, Tottenham, Holloway, Bethnal Green, Hackney, Stoke Newington, and Shoreditch. For each district there is a surgeon. Our own pupils only work in Hoxton, Shoreditch, St. Luke's, and part of Islington, but that is a fairly big district. They find it so in the night when they often have to walk over a mile."

"The outside work is a very valuable portion of the experience gained. In hospital the pupils learn what is required; outside they learn what to do without, or how to make shift. For example, instead of a hot water bottle they generally have to use the oven door and prop it at the feet, and for sheets they usually use newspapers. Many of the patients have not a sheet to their beds."

"Do people never send you sheets or blankets for the poor creatures?"

"Those who know how badly we need them do, and I am sure that many people would help us if they knew to what straits our patients are put—often only having on an old cotton blouse and the husband's coat over them. I can make use of anything from old clothes to blankets."

"How large is your resident staff?"

"I have the assistance of a head nurse, a district midwife, two assistant nurses, and a night superintendent. They have all been here for some time, and their knowledge of the work, and of the unusual conditions under which it is at present carried on, make things much easier than they would be otherwise."

GENERAL TRAINING AND MIDWIFERY.

"Do you take generally trained hospital nurses on special terms?"

"Yes, if they only want to be monthly nurses they can have a month's training for seven guineas. They have to produce the hospital certificate before they can be received. Some come who intend to be generally trained afterwards."

"Which do you think is the better plan?"

"My view is that all girls who propose to be midwives should previously take general training. Personally, I have found general training of the utmost value."

"You were trained in London?"

"At St. Mary's Hospital, Paddington, and I was afterwards sister. I had six years of gynæcological work, and without my general training I should have been quite at sea here."

DIFFICULTIES OF DISTRICT MIDWIVES.

"With your knowledge of the work of district midwives, do you anticipate that there will be difficulties in carrying out the new Act?"

"I do not see how some can be avoided under the stringent provisions of the Act. We have our own doctors to call in if required, but where there is no doctor, who is going to provide one as the Act stipulates? The awful poverty of the women, who, often in our district are found by the pupils lying on sacks, does not seem to have been altogether realised."

"What is your opinion as to a district nurse acting as midwife?"

"I certainly think that a nurse who attends cases of open wounds or sores should not act as midwife. But I do not see why a nurse who only attends medical cases should not do so."

### Central Midwives Board.

### TWO CERTIFICATES CANCELLED.

**ON Thursday, last week, a special meeting of the Central Midwives Board, under the provisions of the Rules of Procedure on the proposed removal of a name from the roll, was held at the Board-room. There were present Dr. Champneys, in the chair; Miss Paget, Mrs. Latter, Dr. Ward Cousins, Mr. Parker Young, and Dr. Cullingworth.**

**The first case heard was that of Margaret Sully, a certified midwife, No. 186 on the roll, against whom the following charges were alleged:—(1) "That on January 29th, 1905, at the Cardiff Police Court, you were convicted of letting lodgings to seamen without being licensed in accordance with the bye-laws of the Corporation of Cardiff in this respect, your application for a license for such purpose having been previously refused on the ground of your bad character;" (2) "That you have harboured women of immoral character."**

**The midwife was present, the charges were read against her, and she was cross-questioned. She admitted having once been fined for letting lodgings without a license, but**

denied all other charges. The Board deliberated on the case in camera, the midwife and the Press were then recalled, and the chairman informed the latter that the Board, having most carefully considered all the evidence, had decided against her, and that her name would be removed from the roll and her certificate cancelled.

The next case was a charge alleged against Hannah Easter Clementson, a certified midwife, No. 1,431:—"That on October 22nd, 1904, while in attendance on Sarah Tromans, of 6 Howe Street, Barrow-in-Furness, then in labour, you were drunk and thereby incapable of performing your duties as a midwife." A letter was read from the midwife explaining her inability to be present and denying the charge; but the written evidence against her was very strong, and it was proved that she had been warned on several occasions. The board deliberated in camera, and when the representatives of the Press were recalled, the chairman read the verdict that this midwife's name would be removed from the roll and her certificate cancelled, at the same time remarking that a midwife who becomes intoxicated is a source of great danger.

This meeting then terminated, and the business of the second special meeting was proceeded with.

The first business on the agenda was the appointment of an inspector. The five candidates selected by the committee were interviewed, and it was finally announced that Dr. Lucy Clapham had been appointed. The issue of advertisement inviting applications for post of examiner was considered, and it was arranged to insert it in the medical papers without delay.

### Novelties for Nurses.

**By Our Shopping Correspondent.**

### CHIVERS JAM.

The name of Chivers as makers of jams, jellies, and other comestibles is so well known that it hardly seems possible to say anything that has not been said in their favour. But it may be not altogether useless to mention that the excellent jams and jellies are to be found at nearly every grocer's, however small, throughout the country, and that they should be especially asked for.

### A RELIABLE NURSE'S WATCH.

Messrs. Joseph Heming, 28 Conduit Street, have ascertained the fact that an unreliable watch is no use to a nurse. Consequently they have devoted themselves to the task of supplying a useful and dependable lever watch for a moderate price, which shall constitute all that a nurse can want. The watch to which I refer is made in silver, gun metal, and gold. It is provided with a seconds dial of proper proportions and the face is clear, and lastly, Messrs. Heming will engrave initials on the case free of cost.

### A NEW NURSES' VEILING.

Messrs. E. and R. Garrould, of Edgware Road, have submitted for inspection a sample of their new "Virnot" veiling. It is dark blue and of an almost perfect texture, having very much the appearance of crêpe de chine. It is much softer and more durable than gossamer, hangs in graceful folds, and is not affected by water. Messrs. Garrould have for some time been trying to manufacture an ideal veiling, and believe that the "Virnot" is the only one which can be placed in water, taken out, dried and ironed, and yet retain all its lustre and softness of appearance. It is quite the nicest nurses' veiling that I have seen, and I have satisfied myself that it will wash and iron without spoiling in the least.

### Appointments.

[No charge is made for announcements under this head, and we are always glad to receive, and publish, appointments. The information, to insure accuracy, should be sent from the nurses themselves, and we cannot undertake to correct official announcements which may happen to be inaccurate. It is essential that in all cases the school of training should be given.]

Bethnal Green Infirmary.—Miss Maude Hexamer and Miss Mary Alice Walmsley have been appointed sisters. Miss Hexamer was trained at the Central London Sick Asylum, Hendon. She has since been at the Lewes Infirmary and done district nursing at Gloucester and private nursing at Eastbourne. Miss Walmsley was trained at the London Hospital and has since been staff nurse.

Hospital St. Cross, Rugby.—Mrs. Dipple has been appointed night charge nurse. She was trained at the General Hospital, Birmingham, and has since been charge nurse in the same institution.

Lewisham Infirmary.—Miss Margaret Rogers has been appointed sister. She was trained at Guy's Hospital, London, and has since been attached to the private nursing staff of that institution. She has also been nurse at the Cancer Hospital, Doncaster.

Llandudno Sanatorium.—Miss Cicely Finnemore has been appointed matron. She was trained at the North Staffordshire Infirmary, Stoke-on-Trent, and has since been housekeeper at Guy's Hospital, London, and at the Derbyshire Royal Infirmary, Derby, and matron at the Free Convalescent Home for Poor Children, Buxton.

Manchester Southern Hospital.—Miss A. M. M. Davies-Brown has been appointed matron. She was trained at the Royal Infirmary, Edinburgh, where she afterwards acted as staff nurse and temporary head nurse. She has since been sister at Manchester Southern Hospital.

St. Mary (Islington), Infirmary, Highgate Hill.—Miss Bessie Williams has been appointed sister. She was trained at Bath United Hospital, and has since been sister at the Hospital for Nervous Diseases, Maida Vale, London.

South Shields Workhouse Infirmary.—Miss Olive M. Blayney has been appointed charge nurse. She was trained at the Chorlton Union Hospital, and has since been nurse at the Royal Infirmary, Preston. She has also done private nursing.

Sudbury Workhouse Infirmary.—Miss Mary Ann Jackson has been appointed superintendent nurse. She was trained at Birmingham Infirmary, where she afterwards became sister.

Throat Hospital, Golden Square, London.—Miss Annie Laura Levan has been appointed staff nurse. She was trained at the East Sussex Hospital, Hastings, where she has since been sister. She has also done private nursing.

Upney (Barking) Isolation Hospital.—Miss Emily Spring has been appointed matron. She was trained at the General Infirmary, Chichester, and was afterwards district nurse. She has since been nurse-matron at an isolation hospital, charge nurse at the North-Eastern Fever Hospital, London, and nurse-matron at the Epsom Isolation Hospital.

### Examination Question for March.

If permitted by the doctor to choose your own means and hours, in what way would you prepare a patient for operation with regard to clearing the bowels?—The Examiner.

### Everybody's Opinion.

[Correspondence on all subjects is invited, but we cannot in any way be responsible for the opinions expressed by our correspondents. No communication can be entertained if the name and address of the correspondent are not given as a guarantee of good faith, but not necessarily for publication. All correspondents should write on one side of the paper only.]

### TRAINED NURSES' ANNUITY FUND.

"Policy No. 5200," Royal National Pension Fund, writes:—With regard to that excellent charity, the Trained Nurses' Annuity Fund, may I plead that all nurses, while providing for themselves in the Royal National Pension Fund, which undoubtedly is the very best way for a nurse to provide for her own old age, should give annually a trifle to the purely charitable fund of the Trained Nurses' Annuity Fund for providing small pensions to those of our less fortunate sisters and co-workers, many of whom started work long before the advantages offered through the Royal National Pension Fund were in existence. Some of us remember how much harder the work was in those days, before comfortable homes were built, or wardmaids were provided for the rough work, and how little off duty was given; and I must add not expected or looked for when there was a "bad case," or any extra pressure in the ward. I appeal to all those who have enjoyed the later privileges, and to the pioneers who went through those hard old days, to help as God has blessed them with health and strength, those poor old, feeble, worked-out sister-nurses by sending some small help to the honorary secretary Dr. Ogier Ward, 73 Cheapside, London, E.C.

### MR. SYDNEY HOLLAND AND REGISTRATION.

"Onlooker" writes: As a trained nurse of some years experience I would like to remark on some of Mr. Sydney Holland's objections to the registration of trained nurses. I cannot quite understand the side he takes, as I have heard that he has always been such a good friend to the trained nurse. He says, "no doctor worth his salt would engage a nurse simply because she was on a register." As nurses I think most of us agree that our training school's recommendation comes first, but what of the "after years," when the matron can only say (with truth) that she can say nothing of the nurse after "the date on which she left her training school." Why do the public mostly turn to the Medical Directory to look up a medical or surgical man's career, on being recommended to go to a fresh one? The only true answer is, I think that both the public and the trained nurse will find the register (when there is one) as useful to them as it has proved to the physician, surgeon, and already, no doubt, to the midwife. Mr. Holland says that his side throws no stones. But he has remarked twice that if a midwife be "so minded she may, when she has finished her case, go and drink herself stupid if she likes" without making any difference to her registration. If I were a midwife I should object to that person belonging to an institution I belonged to; and as there is in existence a Central Midwives Board, I feel sure that, in time, no such "Sairey Gamp" will be allowed on any register. Finally, it is true that a nurse may, as she grows older, get "short-sighted, silly, or even worse." But does such a thing never occur among doctors, lawyers, artists, or even chairmen?

### Practical Hints.

We welcome notes on practical points from nurses.

### ABOUT COFFEE.

1. An ounce of strong black coffee often stops vomiting, and can be kept down when other fluids only cause more sickness; this chiefly applies to anæsthetic sickness.

2. The yolk of an egg beaten and added to coffee and milk makes a nourishing and pleasant drink. Patients who cannot take eggs in other forms often like this combination.

3. A cup of strong hot coffee taken early in the morning acts as a good aperient with some patients, while it often assists where laxatives are required.

4. Coffee is most useful in alcoholic cases; given very strong it lessens the craving for spirits, and in some measure supplies the customary stimulant.

5. As a stimulant after shock it is often ordered to be given per rectum:—*e.g.* strong black coffee, ℥vi.; port wine, ℥ii. Given at temperature 100–105° F.

6. A little coffee burnt on a plate makes an excellent deodorant. The grounds of coffee will clean glass bottles used with plenty of hot water and well shaken.

### TRAVEL NOTES AND QUERIES.

By our Travel Correspondent.

Trip to Bavaria (Ruby North).—If you will give me more particulars we will see if something can be arranged for £10 or £12. To let you have a rough idea as to price I will tell you that the second-class return ticket to Munich *via* Wurzburg is £5 10s. 4d. or *via* Nuremberg £5 14s. 11d. In both cases the ticket will last 45 days. I cannot tell you what your daily expenses will be until I know where you want to go, but roughly speaking, reckoning tips, you cannot do it for less than from 7s. to 8s. per day. At the higher figure that would come to £6 for the 15 days. Besides this you must reckon excursions and incidental expenses. Write me fully and I will arrange the best I can for you.

Cycling in Switzerland (Scotch Tourist).—I do not recommend cycling in Switzerland; my experience is, that very often you are unable to use your machines, roads being unsafe for wheeling, or there being difficulties about forwarding impedimenta, hence the cycle becomes a perfect old man of the mountain, you are compelled to take your burden about with you at considerable cost, which money might have been well spent in carriage hire or trains. It is excessively fatiguing to wheel up the passes and often impossible or very dangerous to wheel down. Give me some idea of where you would like to go. If all is new to you I think I should recommend Meiringen on the Brünig, it is a good centre, within easy reach of the Grimsel, the Schiedegg, Lucerne, Interlaken, Brienz, Thun, and Grindelwald. Second-class return approximately £5 8s. In June hotel charges are much lower than in July and August, and I think you could get good accommodation for five frs. per day. This for a fortnight would come to £2 17s. each, and tips would bring the amount up to about £3 5s., then there would be excursions and incidental expenses. Think the matter over and let me hear again. June is to my mind the ideal month for Switzerland, the meadows and lower mountains are covered with almost all kinds of flowers in rich masses.

Adelboden and Engelberg (Yungfrau).—Very pleased to hear from you again. Adelboden is becoming rather fashionable, but fairly cheap accommodation is still to be had. The Pension Edelweiss offers 5 to 6 francs, except in the height of the season. The same terms at Pension Adler and Pension Hari. Hôtel Pension Wildstrubel from 7½ francs. At Engelberg, Hôtel Pension Engel, terms are from 6½ francs, Hôtel Pension Engelberg, from 6 to 8 francs. Hôtel des Alpes the same. Personally, I should prefer Adelboden. Engelberg is rather enervating in the summer. There are many other charming spots to be considered, but to simple, cheap, out-of-the-way places you usually have to take a long journey. How would you like the little towns round the Lake of Geneva? I could give you a nice address at Lausanne, only on the heights.

Rules in Regard to Correspondence for this Section.—All questioners must use a pseudonym for publication, but the communication must also bear the writer's own name and address as well, which will be regarded as confidential. All such communications to be addressed "Travel Correspondent, 'Nursing Section of *The Hospital*,' 28 Southampton Street, Strand." No charge will be made for inserting and answering questions in the inquiry column, and all will be answered in rotation as space permits. If an answer by letter is required, a stamped and addressed envelope must be enclosed, together with 2s. 6d., which fee will be devoted to the objects of "The Hospital" Convalescent Fund. Ten days must be allowed before an answer can be published.

### Echoes from the Outside World.

### The King's Health.

The King, who has been suffering from a cold, and has therefore remained indoors for a few days, held a Council at Buckingham Palace on Monday, and, among other business, he signed an Order in Council formally giving his approval to the engagement of the elder daughter of the Duke and Duchess of Connaught to Prince Oscar of Sweden and Norway.

### The Queen's Visit to Lisbon.

The Queen was not able to leave Portsmouth until Friday afternoon in consequence of bad weather, and then owing to the rough sea in the Channel the Royal yacht had to seek the shelter of Portland Harbour about 8.30. Her Majesty was able to pursue her journey on Saturday, but on Monday she had to put in at Vigo because of the gale. The last time she was delayed by the weather was in 1868 when, as Princess of Wales, she and the Prince made a State visit to Ireland, and three days elapsed before the gale at Holyhead subsided. On Friday morning the Queen occupied a portion of her time by paying a visit to Gosport, leaving in a naval steam pinnace with Princess Charles of Denmark, and attended by two ladies of the suite; her Majesty called upon a lady who was formerly in the Royal household. The trip was quite private and there was nothing to indicate the rank of the visitors as they walked through the quiet streets.

### Royal Visit to East Ham.

On Saturday afternoon the Prince and Princess of Wales paid a visit to East Ham and formally opened the Technical College and Secondary School which adjoins the municipal buildings at the corner of the High Street. The place was *en fete*, and the day was generally observed as a holiday, most of the houses and shops being decorated. The Prince and Princess were received with enthusiasm on their arrival, and on their way to the Town Hall their attention was directed to a brass tablet on the first landing of the main staircase upon which are recorded the names of about 130 inhabitants of East Ham who were engaged in the South African War, below which was the inscription "Lest we forget." The Prince, in replying to the address welcoming the Princess and himself to the borough, said that it was difficult to realise that only 10 years ago the crowded streets of East Ham were green lanes, that the population had multiplied nearly twentyfold in the last 30 years, and that within the borough one industry alone employs over 10,000 men. His Royal Highness then opened the building, which has been erected at a cost of about £24,000, expressing before he left the satisfaction of himself and the Princess with the whole of the arrangements.

### The War in the Far East.

The Japanese troops have occupied Tie-ling, and an official report states that quantities of provisions and forage were stored in the vicinity of the station, but about two-thirds of them have been burned. It was announced in St. Petersburg, on Friday, that General Kuropatkin had been relieved of the function of Commander-in-Chief, and the position conferred upon General Linevitch. General Kuropatkin was born in 1848, and entered the Russian army in 1864. His career has been one of great brilliance. He distinguished himself under Skobeleff in the campaign 1875; in 1876 he went on a mission to Kashgaria, explored a region until then unknown to European travellers, and wrote an excellent record of his journey; he was Chief of Staff during the Russo-Turkish war; in 1878 he became Governor-General of the Transcaspian Provinces; in 1880 he led the campaign against the Tekke-Turkomans; in 1890 he was gazetted Lieutenant-General; and in 1898 he was appointed Minister of War. There is a strong opinion among military authorities that he has handled the immense forces under him as successfully as any man in Russia could have done in circumstances of extreme difficulty. General Linevitch is ten years older than his predecessor, and his principal claims to distinction are that he rose from the ranks, and led the Russian troops at the relief of Peking in 1900. When war with Japan broke out he was in command of the Russian Army in Manchuria, and had to give way to General Kuropatkin when the latter was nominated Commander-in-Chief. He has now been appointed under General Linevitch to command the First Manchurian Army.

### Centenary of Senor Garcia.

On Friday Senor Manuel Garcia celebrated his 100th birthday. He is an eminent teacher of singing and the inventor of the laryngoscope. During the morning he had the honour of being received by the King who invested him with the insignia of a Commander of the Royal Victorian Order. Later in the day Senor Garcia held a reception of representatives of music and science from all parts of the world. Sir Felix Simon presided, and congratulatory messages were delivered to the Senor from the King of Spain, who conferred on him the Order of Alphonso XII., and from the German Emperor, who bestowed on him the gold medal for science. Addresses and messages of congratulation from several scientific and musical societies, and from Senor Garcia's old pupils, were read, and he was then presented with his portrait subscribed by international contribution and painted by Mr. J. S. Sargent, R.A. In the evening a banquet took place in his honour.

### Factory Disaster in America.

A terrible accident occurred at a shoe factory in Massachusetts, United States, on Monday. A boiler exploded in the building, which was made entirely of wood. The flames spread with great rapidity, so that retreat was almost impossible, and 100 out of 200 operatives in the structure at the time lost their lives. Father Rourke, with the aid of a ladder, which he placed against a window, succeeded in saving the lives of eight girls. He had almost secured a ninth, when suddenly the flames reached him and he was obliged to beat a hasty retreat, leaving the rest of the girls to their sad fate. A policeman at another window was not so fortunate as the priest. He made a gallant attempt to rescue three girls, but they were overpowered and fell back into the flames before he could succeed in reaching them. A man and a girl were found pinned beneath some wreckage in such a manner that only one could escape. The man helped the girl to get free, and lost his own life in consequence.

### Remarkable Prices for Silver Spoons.

At Christie's, last week, a collection of Early English spoons was sold at prices which excited some sensation. A pair which three years ago fetched at the Bone sale £132, realised £265, or £43 per ounce. A set of five Elizabeth Apostle spoons, representing St. James the Less, St. Jude, St. Matthias, St. James the Greater, and St. Matthew, which 12 years ago changed hands for £85, realised no less than £290. Six Maidenhead spoons (Exeter mark), and a master spoon, which were sold at the Bone sale for £36, rose to £49. Of the seal-top spoons one, with a London mark dated 1559, which fetched £39 at the Bone sale, was purchased for £55. Altogether, the sale showed that many silver spoons are now worth more than their weight in gold.

### Notes and Queries.

**FOR REGULATIONS SEE PAGE 328.**

*Fees.*

(184) I should be pleased to know if it is customary for the employer of a permanent nurse to continue her fees during a month's holiday in the year, especially in a case where a substitute is necessary?—*Dott.*

It would entirely depend upon the terms arranged at the time of the engagement, or the generosity of the employer. A yearly engagement usually carries with it the privilege of a holiday.

*Deafness.*

(185) Where can I procure an audiphone?—*A. B.*

Mr. T. Hawkesley, 357 Oxford Street, W. It was invented by Mr. Rhodes, of Chicago, U.S.A.

*Registration and Books.*

(186) 1. Will you kindly tell me where I should write as regards being registered? I finished my general training, and am now a certificated nurse. 2. Can you also tell me in what paper to advertise, or where is the best place to sell medical books?—*Nurse C.*

1. There is no registration of nurses. 2. Medical books are difficult to sell. We do not advise you to advertise. Apply to a good bookseller.

*America.*

(187) Will you kindly inform me the best means of obtaining an engagement as nurse in the Northern States of America? I should prefer a post in hospital if possible; otherwise, to become connected with some private nursing institution.—*D. H.*

See "The Nursing Profession: Where and How to Train," which is probably in the library at your hospital.

*Swiss Nurse.*

(188) I should be glad if you could inform me if any of the London or provincial hospitals would take a Swiss nurse (speaking English) for a short time? She was trained at the Zurich Hospital?—*M.S.M.*

Write to some, or an advertisement in The Hospital might have the desired result.

*Certificate.*

(189) I have had three years' asylum training, and have done three years' private nursing. Since then I had one and three-quarters year's training in a London hospital. I gave it up as my health was so completely run down, and I did not then feel equal to finishing my three years. As they will not now take me back again to finish my training, could you tell me of a good hospital where I could enter and get a recognised certificate without giving up another three years, so that I could get a good post afterwards, and, in case of registration, would be qualified.—*One in Difficulty.*

Excepting by returning to the hospital where you had one and three-quarters year's training, you could not nowadays get a recognised certificate for general nursing without giving up another three years.

*Poison Doses.*

(190) Will you kindly tell me the name of a book on dispensing with the average dose of, and antidote to, the various poisons?—*Nurse Matron.*

"What to do in Cases of Poisoning." By S. Murred. Price 3s. 6d. It can be obtained from the Scientific Press, 28 & 29 Southampton Street, Strand, W.C., or through any bookseller.

*Convalescent Home.*

(191) Can you tell me of any convalescent home where a young man, living in London, could be received after having inflammation of the lungs. He is very poor and cannot pay much?—*Poverty.*

See "Burdett's Hospitals and Charities," the Scientific Press, 28 & 29 Southampton Street, Strand, W.C.

*Private Hospital for Infectious Cases.*

(192) I am contemplating opening a small private hospital, on my own account, for infectious cases; but, before doing so, would be glad to learn whether you know of any obstacle in the way of carrying out such a project.—*I. M.*

You must consult the local authorities and obtain their permission. If it be given, and providing you have sufficient capital, the support of the medical men in the town, and the requisite experience and capacity for the management of such an undertaking, you might succeed in one of the large cities.

**Handbooks for Nurses.**

| | Post Free. |
|---|---|
| "A Handbook for Nurses." (Dr. J. K. Watson.) ... | 5s. 4d. |
| "The Nurses' Dictionary of Medical Terms." ... ... | 2s. 0d. |
| "Massage" (Swedish.) ... ... ... ... ... ... | 2s. 6d. |
| "Surgical Ward Work and Nursing" ... ... ... ... | 3s. 10d. |
| "A Complete Handbook of Midwifery." (Watson.) ... | 6s. 4d. |

Of all booksellers or of The Scientific Press, Limited, 28 & 29 Southampton Street, Strand, London, W.C.

### For Reading to the Sick.

"STAY THOU NEAR ME."

Stay near me through this heat and glow,

Stay near, too, when my day sinks down,

And long the evening shadows grow,

And the night comes stealing on.

Lay in blessing, then, Thy hand

On my weary, weakly head;

Saying, "Rest, child! to the land

Thy faith hath sought thou shalt be led.

Stay near me; in Thine arms enfold,
When most the chill of death I dread;
Chill, like the sharp and bitter cold,
Ere dawns in Heaven the morning red.

When darkness shall mine eyes o'ertake,

Light Thou my spirit through the gloom,

That unto me the morn may break

As breaks to him the exile's home.

*From the German of T. W. Spitta.*

There are times when a dense cloud veils the sunlight: you cannot see the sun, nor feel him—sensitive temperaments feel depression; and that unaccountably and irresistibly. No effort can make you feel. Then you hope. Behind the cloud there is the sun; from thence he will come: the day drags through, the darkest and longest night ends at last. Thus we bear the darkness, and many a sleepless night. It does not shine now, but it will.

So, too, spiritually. There are hours in which physical derangement darkens the windows of the soul; days in which shattered nerves make life simply endurance; months and years in which intellectual difficulties pressing for solution shut out God. There are moments of a hopelessness, when our highest feelings have been misunderstood and our purest met with ridicule; times when our heavy secret was lying unshared like ice upon the heart.

And then the spirit gives way: we wish that all were over; that we could lie down tired, and rest like children from life; that the hour was come when he could put the extinguisher on the lamp, and feel the last grand rush of darkness on the spirit. Then faith must be replaced by hope. "What I do, thou knowest not now, but thou shalt know hereafter." "Clouds and darkness are round about Him, but righteousness and truth are the habitation of His throne."—*F. W. Robertson.*

Never let us be discouraged with ourselves; it is not when we are conscious of our faults that we are the most wicked; on the contrary, we are less so. We see by a brighter light; and let us remember, for our consolation, that we never perceive our sins till we begin to cure them.

*Fénelon.*

Make me Thine own

And take me:—of myself I am afraid

Oh, take me from myself; oh, take away

Whate'er of self is in me; and I pray

Give me on what my spirit may be stayed,

And that, I know full well, is but

Thyself alone.

*T. Williams.*

